# Long-Lasting Simultaneously Hydrophilic/Oleophobic Behavior of a Nanometer-Thick Ionic Liquid Coating

**DOI:** 10.1021/acs.langmuir.5c03361

**Published:** 2025-12-02

**Authors:** Alan Tirado, Newt Kouma, Anumita Kumari, Fajer Almanea, Meghan Vander Woude, Deirdre Kelly, Haitao Liu, Lei Li

**Affiliations:** † Department of Chemical & Petroleum Engineering, 6614 University of Pittsburgh, Pittsburgh, Pennsylvania 15261, United States; ‡ Department of Chemistry, University of Pittsburgh, Pittsburgh, Pennsylvania 15260, United States

## Abstract

Surfaces more wettable to water than to oil are highly desirable for applications such as oil/water separation, detergent-free cleaning, and antifogging coatings. However, such surfaces are rare, typically relying on fluoropolymer coatings requiring multistep modification, and often lose oleophobicity with time, limiting their practical use. Ionic liquids (ILs) are promising alternatives due to their thermal and chemical stability and highly tunable structures. Here we report a functionalized ionic liquid coating with highly fluorinated alkyl segments and polar end-groups. This nanometer-thick coating achieves strong oleophobicity on silica substrates, while maintaining a water contact angle (WCA) below 10°. Importantly, the oleophobicity remains stable over extended exposure, reflecting robust wetting behavior attributed to the coating’s rigid structure and limited chain relaxation. We further demonstrate that the coating enables long-term antifogging and detergent-free cleaning. These results highlight the potential of functionalized ionic liquids to address the challenge of creating surfaces with controlled, unconventional wettability.

## Graphical abstract

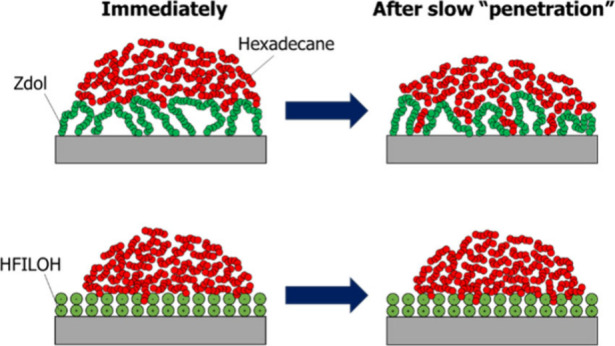

## Introduction

The vast majority of solid surfaces, whether natural or manmade, are more oleophilic than they are hydrophilic; that is, they are more wettable by oils than by water.^1–4^ However, surfaces with wettability differing from conventional materials hold considerable appeal across a range of applications. Although many specialized surfaces and coatings have been developed, there remains a demand for materials that exhibit a greater affinity for water than for oils. Achieving simultaneous hydrophilic and oleophobic properties remains challenging because lower surface tension liquids like oils naturally we most surfaces more effectively than higher surface tension liquids such as water.^1–6^ The scarcity of surfaces and coatings demonstrating this dual property presents a technological void, particularly for long-lasting antifogging and detergent-free cleaning applications.^3,7,8^ Another important application is oil/water separation, where membranes with hydrophilic/oleophobic properties offer passive and highly selective water removal capabilities.^3,9–13^

Hutton et al. were among the earliest to report hydrophilic/oleophobic surfaces, achieved using a cationic fluorosurfactant complexed with a weakly negatively charged plasma polymer surface, yielding a water contact angle (WCA) below 20° with a hexadecane contact angle (HCA) at 82°.^3,14^ They attributed this behavior to monolayer reorganization that allowed water to interact with the underlying hydrophilic surface. Since then, many reported systems have relied on fluoropolymers—such as polyethylene glycol with fluorinated end-caps (f-PEG),^15,16^ polydicotylfluorine (PFO),^17–19^ perfluoropolyether (PFPE),^1,2,6,20^ and polyvinylidene fluoride (PVDF)^21,22^ —as major components. While fluoropolymers are attractive for their chemical stability, their inherently low surface energy promotes nonselective repellency toward both polar and nonpolar liquids, making it challenging to achieve selective wettability without additional modifications. To address this, many strategies have incorporated copolymeric substituents such as poly­(diallyldimethyl­ammonium chloride)^17,18,23,24^ or solid loadings including SiO_2_ ^17–19,25^ and CaSi_2_ ^26^ nanoparticles. Other groups have focused on hydrophilic/oleophobic membranes, typically employing supports such as cellulose or steel mesh that are subsequently modified with coatings like silanes or hydrogels.^9–12,18,27–29^ For example, Xue et. al created a superhydrophilic and superoleophobic filter by grafting polyacrylamide hydrogel onto stainless steel mesh, achieving underwater–oil contact angles over 150° and over 99% separation efficiency for oil/water mixtures.^12^ Similarly, Chi et. al coated polyester fabric with a monomer solution of 1H,1H,2H,2H-tridecafluoro-n-octyl acrylate, methacrylic acid, and polyethylene glycol diacrylate, followed by UV polymerization, producing a covalently grafted copolymer layer that displayed oil contact angles near 150° along with notable abrasion resistance.^30^

Previous studies in our group focused on single-component perfluoropolyether (PFPE) coatings such as Z-03, Zdol, and Ztetraol, investigating the effects of polar end groups.^1,2^ These results indicated that the degree of hydroxyl functionalization is crucial for hydrogen bonding between PFPE and the native oxide layer of Si wafers, which in tern governs selective wetting. In the absence of hydroxyl groups (Z-03), the coated surface remained hydrophilic and oleophilic, consistent with disordered chain arrangements that allowed both water and hexadecane to permeate. At the opposite extreme, excessive hydroxyl content (Ztetraol) produced dense packing through strong hydrogen bonding, yielding surfaces that were hydrophobic and oleophobic. With intermediate hydroxyl content (Zdol), however, the PFPE chains packed loosely enough to admit small water molecules while hindering the penetration of bulkier hexadecane. Time-dependent studies further supported this mechanism: the oleophobicity of Zdol degraded upon oil exposure, as the high HCA reflected a kinetically trapped state rather than the thermodynamic equilibrium.^1–3^ The mobile polymer chains gradually relaxed, allowing hexadecane to infiltrate and eventually reach the underlying surface, while water penetrated immediately due to its smaller size. This instability of oleophobicity significantly limits the practical application of these previously reported hydrophilic/oleophobic coatings.

Ionic liquids are promising candidates for further exploration of specially wettable surfaces. Their strong thermal and chemical stability, coupled with highly tunable molecular structures, has enabled their use in diverse applications over the past few decades.^31,32^ For surface coatings, low surface tension is desirable to achieve uniform wetting. This property can be tuned by incorporating short fluorinated alkyl segments into the ionic liquid structure.

In this work, we expand on our previous studies by investigating a nanometer-thick functionalized ionic liquid coating featuring highly fluorinated alkyl segments and polar end groups. This coating exhibits simultaneous hydrophilic and oleophobic behavior under ambient conditions, with a water contact angle below 10° and a hexadecane contact angle of approximately 70° on silica substrates. Unlike most reported coatings that rely on fluoropolymeric architectures, microscale thicknesses, or underwater conditions, this behavior is achieved with a single, molecularly thin dip coating step. Although the ionic liquid contains polyfluoroalkyl chains, the extremely small amount of material required minimizes the potential environmental and health risks associated with polyfluoroalkyl substances (PFAS). Moreover, the oleophobicity remains stable under continuous oil exposure for at least 48 h, indicating resilient surface organization. Additionally, we qualitatively demonstrate antifogging and detergent-free cleaning capabilities, highlighting the coating’s potential for multifunctional surface applications.

## Experimental Section

### Preparation of Samples

The synthesis of HFILOH has been described previously.^33^ The IL nanofilms were applied to the Si wafer surface via a dip-coating procedure previously established in our lab.^34^ The HFIL–OH was dissolved in Vertrel XF to prepare dilute solutions; Vertrel XF was selected for its high volatility and ability to fully dissolve the ionic liquid at room temperature. Substrates undergo 30 min of UV/Ozone treatment using a BioForce Nanosciences UV/Ozone Procleaner (Power specifications: 110 V AC, 50/60 HZ, 0.5A, and 1 PH) with 185 and 254 nm wavelengths in ambient air at room temperature. The substrate is vertically submerged into and withdrawn from the solutions at 1 mm/s using a KSV Instrument dip coater. Film thickness was controlled by solution concentration: 0.125 g/L yielded ∼0.65 ± 0.07 nm, 0.3 g/L yielded ∼0.85 ± 0.07 nm, and 0.5 g/L yielded ∼1.45 ± 0.07 nm on Si wafers.

Thicknesses of the fabricated IL nanofilms are measured using a J. A. Woollam alpha-SE Spectroscopic Ellipsometer at an incident angle of 75° and a beam diameter of ∼2 mm. Optical constants of the native oxide surface are determined using ″NTVE_JAW″ database complex refractive index after UV/Ozone treatment and prior to dip coating. After dip coating, film thicknesses were extracted using the Cauchy dispersion model. Reported values represent the average thickness within the beam spot. Thickness measurements could not be obtained on glass substrates because their transparency prevented adequate reflection of the ellipsometer beam, but all experiments run on glass slides used the 0.5 g/L dip coating solution.

### Contact Angles

The HCA and WCA of nanometer-thick lubricants on the substrates were measured using a VCA Optima Contact Angle system. 0.5 μL drops of testing liquids were automatically dispensed, and the drop shapes were recorded using a CCD camera and analyzed using vendor-supplied software. Reported values represent averages from at least three substrates with similar film thicknesses (within 0.15 nm). To minimize hydrocarbon contaminations, sample substrates were confined in an acrylic container sealed with Parafilm for the duration of each experiment.

Advancing contact angles were measured by dispensing 0.5 μL drops and then raising the stage until the syringe needle entered the drop, after which up to 1 μL of additional liquid was dispensed while continuously imaging. Measurements were taken from the frame immediately before the drop baseline expanded. Receding contact angles were measured by dispensing 1.5 μm drops and then withdrawing up to 1 μL of liquid through the needle while continuously imaging. Measurements were taken from the frame just before the drop baseline began to contract. Due to the amphiphilic nature of the Si native oxide surface, advancing and receding contact angles could not be obtained on uncoated Si wafers. Similarly, advancing and receding angles could not be obtained on the HFILOH coated Si wafer, even at multilayer thickness.

### X-ray Photoelectron Spectroscopy

Monolayer and multilayer film were analyzed using a ThermoFisher EscaLab 250Xi photoelectron spectrophotometer. Monochromatic Al Kaf X-ray was used as the source of the incident radiation. The pressure of the analysis chamber was 2 × 10^–9^ mBar. Pass energy was 150 eV for survey spectra and 20 eV for fine spectra of N 1s and C 1s. Dwell time was 50 ms for survey spectra and 100 ms for fine spectra of N 1s, F 1s and C 1s. Step length was 0.1 eV for survey spectra and 0.07 eV for fine spectra. The measurements were taken at 0° and 45° for both monolayer and multilayer film. CasaXPS was used to fit the peaks. Shirley background was used and Gaussian–Lorentzian 50% was used to fit the curve. The aliphatic carbon peak was set to 284.6 eV and the binding energies were corrected.

### Antifogging

Glass slides were taken as-is from the packaging and dip coated according to the aforementioned procedure. The coated slides were then laid out on the benchtop beside uncoated control slides and both sets were exposed to the ambient environment. After 3 weeks, slides from each set were held 1 in. above boiling water for 10 s to allow sufficient condensation to form. The slides were then immediately held 1 in. above a self-written sample text to qualitatively observe distortion.

### Detergent-Free Cleaning

Glass slides were taken as-is from the packaging and dip coated according to the aforementioned procedure. The coated slides were then laid out on the benchtop beside uncoated control slides and both sets were exposed to the ambient environment. After 3 weeks, several drops of red-dyed hexadecane were deposited on the surface of slides from each set. The slides were then immediately hand-dipped twice into clean DI water before collecting images.

## Results and Discussion

Simultaneously hydrophilic and oleophobic behavior is demonstrated using a hydroxyl-functionalized, highly fluorinated Ionic Liquid (HFILOH), i.e. 1–1H,1H,2H,2H-perfluorohexyl-3–2-hydroxylethylimidazolium bis­(nonafluorobutanesulfonyl)­imide, recently developed in our lab,^33^ coated onto silicon wafers at nanometer-scale thicknesses. The molecular structure can be seen in Figure 1. Previous work^33^ has shown that the monolayer (ML) thickness of HFILOH is approximately 0.8 nm based on step changes in surface roughness observed by atomic force microscopy as well as molecular dynamics simulations. A clean silicon wafer is both highly hydrophilic and highly oleophilic, exhibiting very low contact angles (∼0°) for both water and hexadecane. Figure 2 compares the initial WCA and HCA of ∼ 1.45 nm HFILOH-coated Si wafers with those of the bare wafers. After applying the HFILOH coating above monolayer thickness, the initial WCA remains low at 7.1°, while the HCA increases significantly to 72.4°. Dynamic contact angle results are summarized in Table 1. For hexadecane on the coated wafer, the receding angle is 64.3° while the advancing angle is 77.1°, corresponding to a relatively low hysteresis of 12.7°, which suggests that the surface is uniform from the perspective of the hexadecane. Dynamic advancing/receding angles could not be measured for the bare Si wafer, or for water on coated Si wafers, because the strongly wetting nature of these surfaces caused the drops to spread continuously.

**Figure 1:**
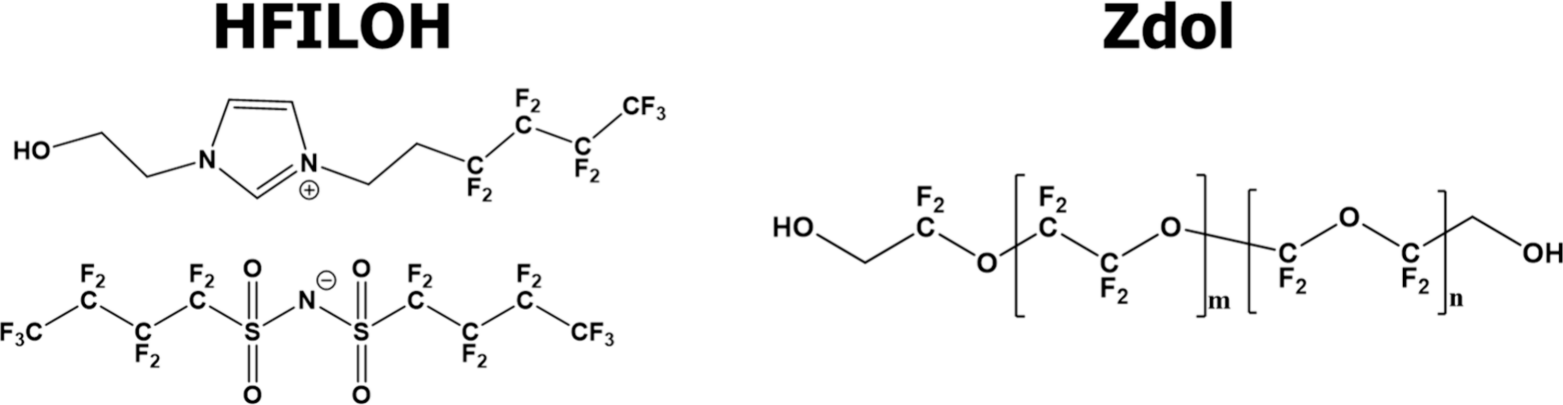
Chemical structures of HFILOH and Zdol. The long polyether backbone of Zdol allows higher conformational mobility, whereas the short alkyl segments in HFILOH confer greater rigidity.

**Figure 2:**
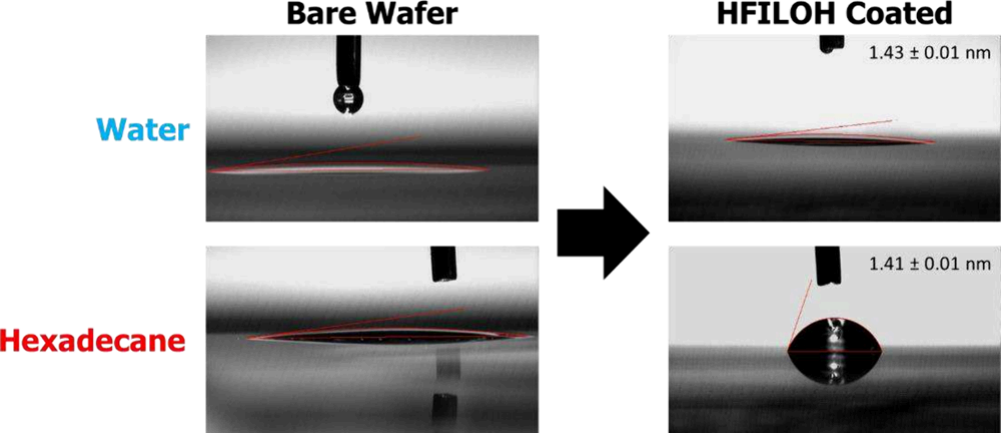
Comparison of deposited water and hexadecane drops on Si wafers before and after coating with ionic liquid.

**Table 1:** Dynamic Contact Angles and Hysteresis

	Receding WCA	Static WCA	Advancing WCA	Hysteresis
Bare Si Wafer	-	∼0	-	-
Coated Si Wafer	-	7.1° (±1.2°)	-	-
Bare Glass Slide	7.2° (±0.2°)	7.5° (±0.7°)	11.2° (±0.2°)	4.0° (±0.3°)
Coated Glass Slide	9.2° (±0.3°)	11.7° (±0.5°)	16.2° (±1.7°)	6.9° (±1.7°)

	Receding HCA	Static HCA	Advancing HCA	Hysteresis
Bare Si Wafer	-	∼0	-	-
Coated Si Wafer	64.3° (±0.5°)	72.4° (±1.9°)	77.1° (±0.1°)	12.7° (±0.5°)
Bare Glass Slide	5.6° (±0.3°)	12.4° (±0.5°)	15.4° (±1.0°)	9.7° (±1.1°)
Coated Glass Slide	62.8° (±2.2°)	63.9° (±0.3°)	76.1° (±1.7°)	13.4° (±2.8°)

To observe the time-dependence of a single hexadecane drop on the HFILOH coating, the drop was deposited onto the substrate in a closed environment, limiting potential contamination due to ambient hydrocarbons in the air. Figure 3a shows that on HFILOH-coated silicon wafers, the HCA only decreases by only ∼3° within the first two hours. As shown in Figure 3b, it then gradually levels off, decreasing by only an additional 2° over the next 46 h, demonstrating that the coating’s oleophobicity is resilient under constant oil exposure. In contrast, on a Zdol-coated wafer, the HCA starts at 69.8° ± 1.0° and drops to 57.6° ± 3.6° within the first two hours. After 48 h, it further decreases to 49.2 ± 3.0° and shows now leveling with over time. The reductions in HCA with time are likely caused by a relaxation interaction between the hexadecane and the coating materials, as a typical solid surface such as polytetrafluoroethylene (Figure S1, Supporting Information) generally maintains a constant HCA with respect to time. The greater stability of HFILOH is likely due to its inherently more rigid structure compared to that of Zdol. As shown in Figure 1, the Zdol coating consists of flexible polyether chains that can more easily rotate and rearrange, allowing hexadecane to penetrate with enough time. The smaller and more rigid cation and anion of HFILOH, however, allow only minimal relaxation initially, which is largely inhibited thereafter. To complement the oleophobic measurements, the time-dependence of the WCA on HFILOH was also studied. Over the measurable lifetime of a water drop (∼1 h in a humid environment) the WCA remained steady at 6.4° ± 1.4° (Figure S2, Supporting Information), indicating that the hydrophilic behavior is stable under these conditions.

**Figure 3:**
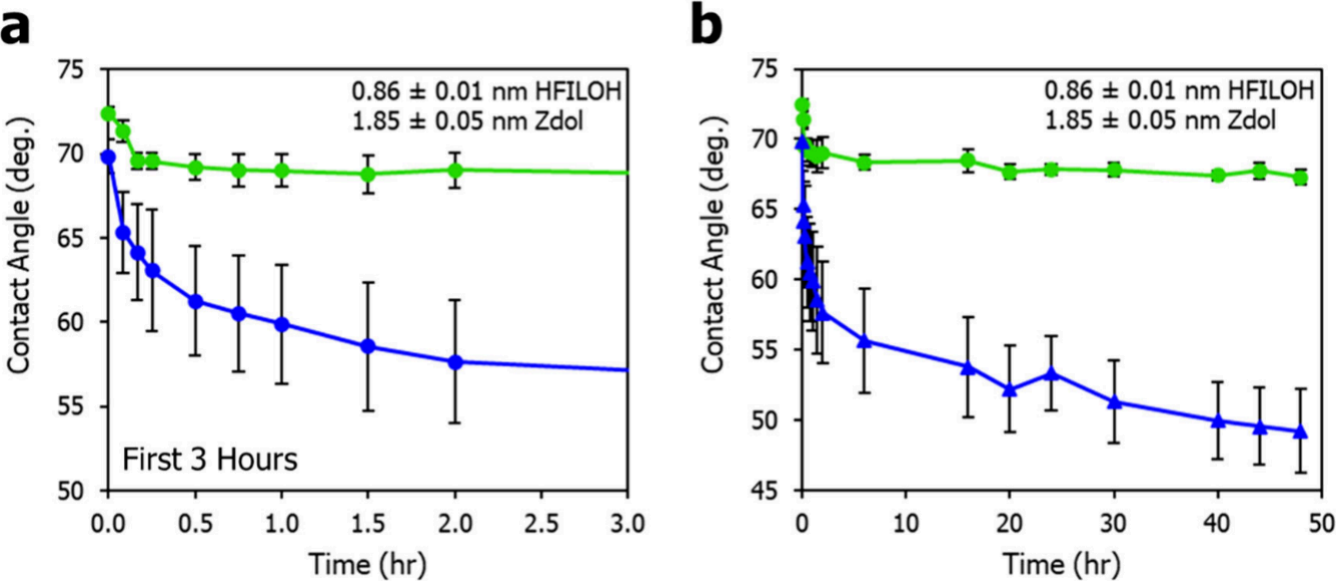
Same-drop HCA measurement on HFILOH-coated Si wafer compared to Zdol-coating with (a) early behavior under 3 h and (b) long-term behavior up to 48 h.

Figure 4 compares HCA values for coating of different thicknesses, demonstrating that thicker coatings exhibit greater oleophobicity than thinner ones. Notably, there is a clear difference between coatings above and below the ML thickness for HFILOH. For the two thicknesses above ML, the coating stabilizes after an initial relaxation during the first two hours. However, the HCA of the thinner coating continues to decrease over time, resembling the behavior observed for Zdol. This behavior may result from incomplete coverage below the ML, allowing the hexadecane to interact more readily with the underlying Si wafer over time.

**Figure 4:**
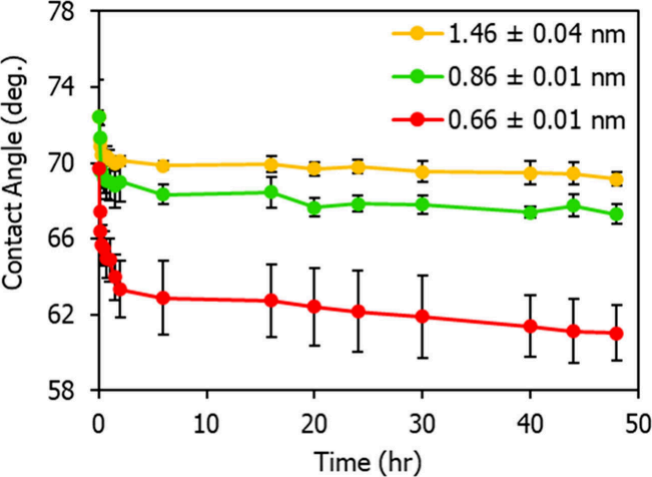
Comparison of time-dependent HCA for HFILOH coatings below, near, and above the ML thickness of ∼0.8 nm.

The intrinsic stability of the coating was evaluated to confirm that the observed behaviors are not due to sample aging. Instead of tracking the contact angle of a single drop over time, new drops were applied to different locations on the same substrate at various time points, and the initial contact angles were recorded. As seen in Figure 5, although the initial WCA and HCA vary slightly at each time point, the variations are random and show no systematic trend over time. These results indicate that the coating properties remain stable, and that the small decreases in HCA for a single drop are attributable to relaxation interactions with hexadecane, rather than coating aging. Similar behavior is observed for WCA, which averages 7.5° ± 2° over the first 48 h postcoating.

**Figure 5:**
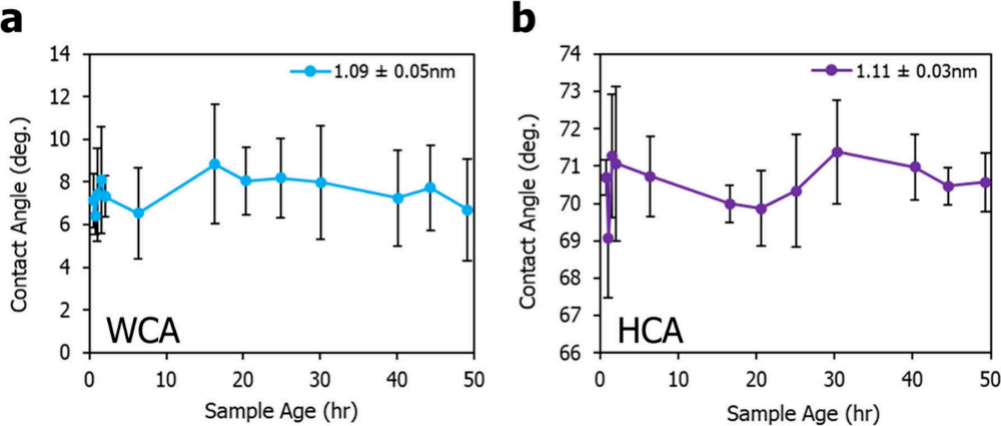
Aging behavior of the HFILOH coating as measured by (a) initial WCA and (b) initial HCA. Each data point represents new drops of liquid on the aged sample.

To investigate the molecular orientation of HFILOH coatings and its impact on wetting behavior, angle-resolved X-ray photoelectron spectroscopy (ARXPS) spectra of C 1s and N 1s for were collected for coated silicon wafers and are shown in Figure 6. Peak positions and areas can be found in Table S1 in the Supporting Information. For monolayer-thick coatings (0.88 ± 0.01 nm), the ratio of CF_2_/CF_3_ peaks to nitrogen peaks increases significantly at a 45° incident angle relative to 0°. Since the 45° measurement is more surface sensitive, this indicates that the fluorinated carbon chains preferentially occupy the coating/air interface, whereas the cationic imidazole ring and the anionic sulfonimide group reside deeper within the film. The fluorinated segments positioned at the surface likely contribute largely to the observed oleophobicity. When the same measurement is taken on a thicker coating (1.32 ± 0.01 nm), the peaks ratios at both angles are nearly identical. This behavior can be explained by a dewetting mechanism, in which IL deposited beyond the monolayer thickness forms domains resembling bulk material rather than an ordered film.^35^ Nevertheless, the coating remains highly oleophobic and hydrophilic, as the fluorinated segments likely remain at the local surfaces of the dewetted domains, albeit in a less uniformly ordered arrangement than in the smooth monolayer.

**Figure 6:**
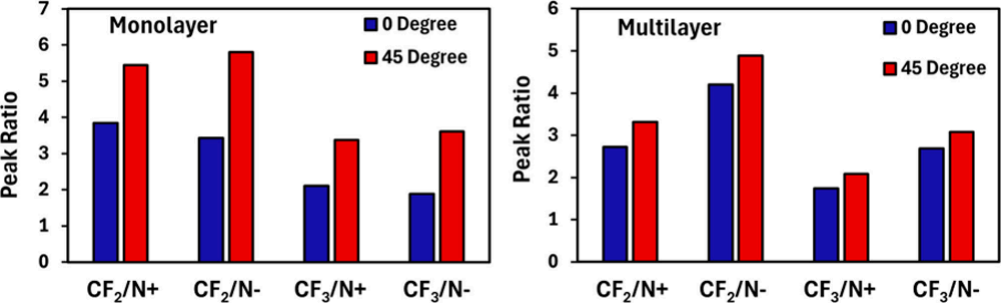
Comparison of ARXPS peak ratios between fluorinated C 1s peaks and each N 1s spectra for HFILOH coatings at and above monolayer thickness

In addition to its long-term stability, the HFILOH coating remains reusable even after full immersion in water. Immersion maximizes water penetration into the coating, which could compromise performance if the liquid were trapped or dissolved the material. To assess this, a silicon wafer coated with a 0.90 ± 0.02 nm HFILOH layer was submerged in deionized water for 60 s and left to dry under ambient conditions for 50 min. After drying, the coating thickness decreased to 0.72 ± 0.06 nm, indicating a partial loss of material. Contact angle measurements shown in Figure 7 reveal a ∼12° decrease in HCA and ∼6° increase in WCA relative to the pristine surface. While this reflects a modest reduction in performance, the coating clearly retains its hydrophilic/oleophobic behavior and is reusable without reapplication or special treatment. The high residual thickness after immersion indicates strong physical adhesion to the substrate, even in the absence of covalent bonding.

**Figure 7:**
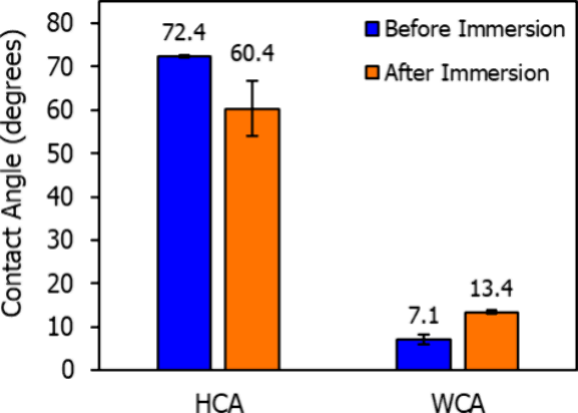
Hexadecane and water contact angles on a silicon wafer with a 0.90 nm HFILOH coating before and after immersion in deionized water for 60 s.

Some practical applications of this hydrophilic-oleophobic coating have also been demonstrated. Glass was chosen as the substrate for these demonstrations because, like Si wafers, it is a polar material, but it also offers transparency that makes it well-suited for optical tests and improved visibility. Dynamic contact angle measurements on coated glass, shown in Table 1, confirm that the coating produces similarly low hysteresis as on Si wafers, indicating robust wetting behavior and uniform surface chemistry. Fogging occurs when water condenses as tiny beads on hydrophobic surfaces, scattering light. On a hydrophilic surface, water forms smooth films, reducing light scattering.^8^ Although clean glass is naturally hydrophilic, surface hydrocarbon contamination gradually increases its hydrophobicity. Similarly, superhydrophilic or superhydrophobic antifogging coatings are often susceptible to contamination by lower surface tension liquids.^8^ Figure 8 shows two initially clean glass slides exposed to ambient conditions for 3 weeks. The HFILOH-coated slide exhibits only minor light distortions in areas of excessive water accumulation, whereas the uncoated slide shows reduced optical transmittance across its surface.

**Figure 8:**
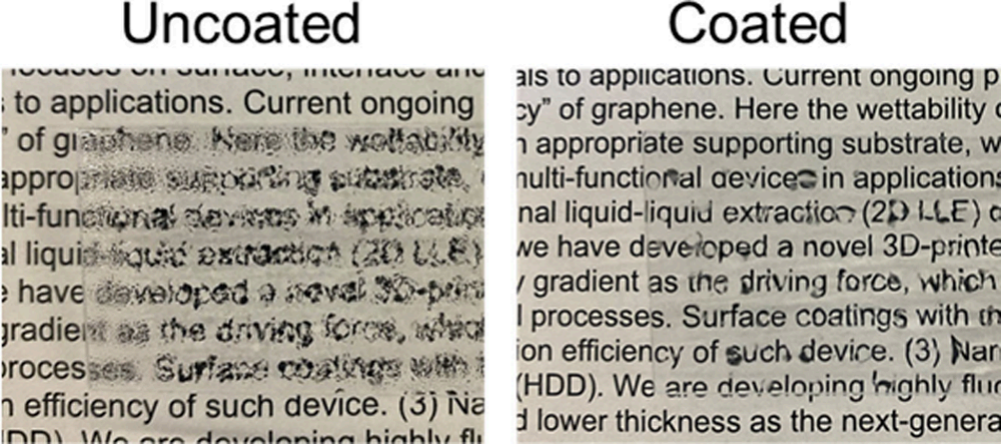
Glass slides have been aged for 3 weeks of contamination before exposure to steam to promote fogging. The HFILOH-coated slide shows significantly improved visibility compared to the bare slide.

Another notable application is detergent-free surface cleaning. Because most surfaces are more readily wetted by oils than water, water alone cannot effectively displace oils from soiled surfaces. Traditional cleaning methods rely on surfactants, which interact with hydrocarbons to enable their removal by water, but these are often environmentally harmful. Figure 9 depicts two slides that were exposed to ambient conditions for 3 weeks, followed by the deposition of several drops of dyed hexadecane. On the uncoated slide, the drops immediately spread and wetted large areas of the surface. In contrast, on the coated slide the drops retained compact bead shapes and did not spread, despite similar volumes. Subsequent immersion in water further demonstrated that the coated slide remained more hydrophilic than oleophilic. Significant oil residue remained on the uncoated slide, as expected for ordinary surfaces without surfactants. In contrast, the HFILOH-coated slide appeared completely clean, indicating that water alone displaced the oil and wetted the surface.

**Figure 9:**
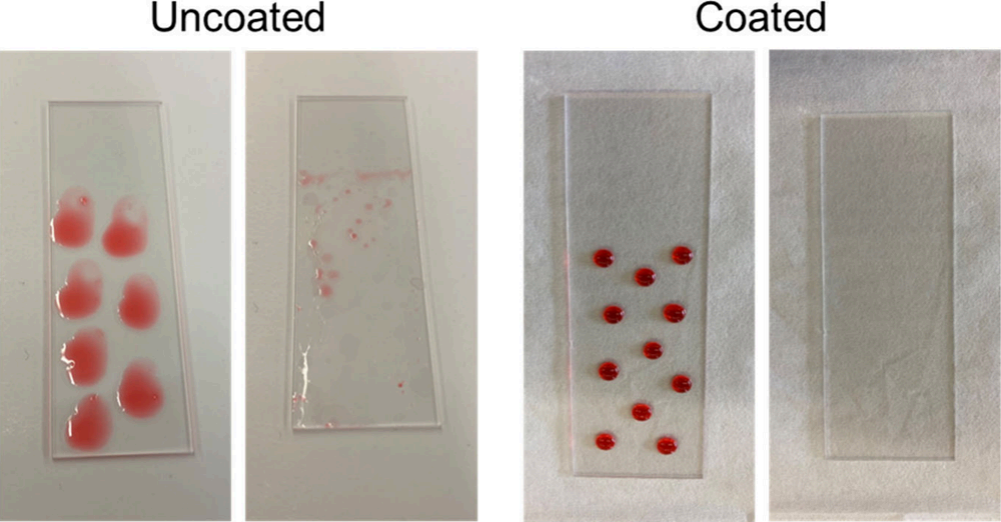
Self-cleaning of hexadecane from uncoated (left) and HFILOH-coated (right) glass slide after 3 weeks of ambient contamination.

## Conclusion

In conclusion, highly hydrophilic and oleophobic surfaces were successfully created using an imidazolium-based ionic liquid with highly fluorinated alkyl segments and hydroxyl functionalization. A facile dip-coating procedure produced nanometer-thick coatings using minimal material while achieving effective and stable water and oil wetting properties. Time-dependent experiments showed that after the HCA initially reduced by a couple degrees, oleophobicity remained strongly maintained over extended exposure. This high stability can be attributed to its inherently rigid structure and limited chain relaxation. Contamination tests further reinforced these findings, as the coated glass slides retained their hydrophilic/oleophobic behavior even after 3 weeks of ambient exposure. Total immersion tests demonstrated that the coating remains reusable, though additional immersion cycles and abrasion resistance experiments are needed to further validate its suitability for practical application. While XPS results suggest that the molecular arrangement favors fluorinated segments at the surface-air interface, further mechanistic studies and optimization may enable broader application of ionic liquid coatings with tunable wettability.

## Supplementary Material



## Notes

Additional experimental details and methods, including auxiliary data that support the conclusions but may crowd the main text (PDF)
