# Supplementary material for: Long-Lasting Simultaneously Hydrophilic/Oleophobic Behavior of a Nanometer-Thick Ionic Liquid Coating
Source: Langmuir. 2025 Dec 2;41(49):33139–45. doi: 10.1021/acs.langmuir.5c03361 (PMC12713771; doi:10.1021/acs.langmuir.5c03361)
Supplement: Supplementary file 1 [file la5c03361_si_001.pdf]

Supporting Information: **Long-Lasting Simultaneously Hydrophilic/Oleophobic Behavior of a Nanometer-Thick Ionic Liquid Coating**

Alan Tirado[a], Newt Kouma[a], Anumita Kumari[b], Fajer Almanea[a], Meghan Vander Woude[a], Deirdre Kelly[a], Haitao Liu[b], Lei Li[a]**

(*: Corresponding Author)

[a] Department of Chemical & Petroleum Engineering, University of Pittsburgh,

Pittsburgh, Pennsylvania 15261, United States

*E-mail: lel55@pitt.edu

[b] Department of Chemistry, University of Pittsburgh,

Pittsburgh, Pennsylvania 15260, United States

*E-mail: hliu@pitt.edu

Number of pages: 5

Number of figures: 5

Number of schemes:0

Number of tables: 6

Table of Contents

Additional Contact Angle Data……………………………………………………………S2

XPS Data...……………………………………………………………………………S3

AFM Data……………………………………………………………………………..S5

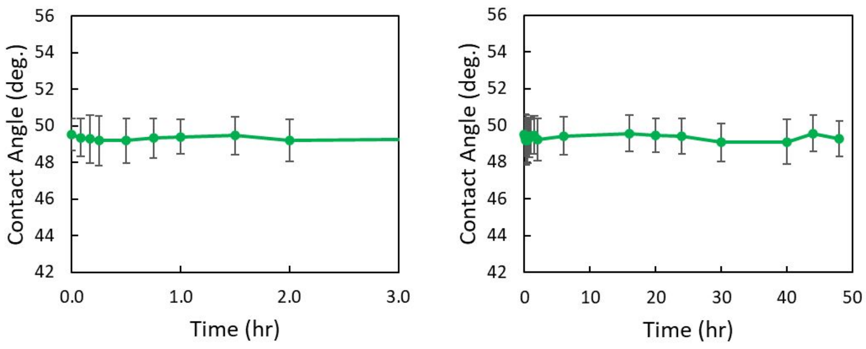

Figure S1: HCA on Bare Teflon substrate. Contact angle is completely static for the duration of 48 hours

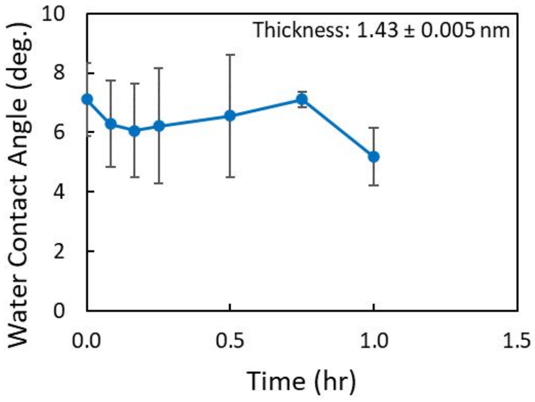

Figure S2: Time-dependent WCA on HFILOH/Si wafer. Contact angle remains relatively static for the measurable lifetime of drop

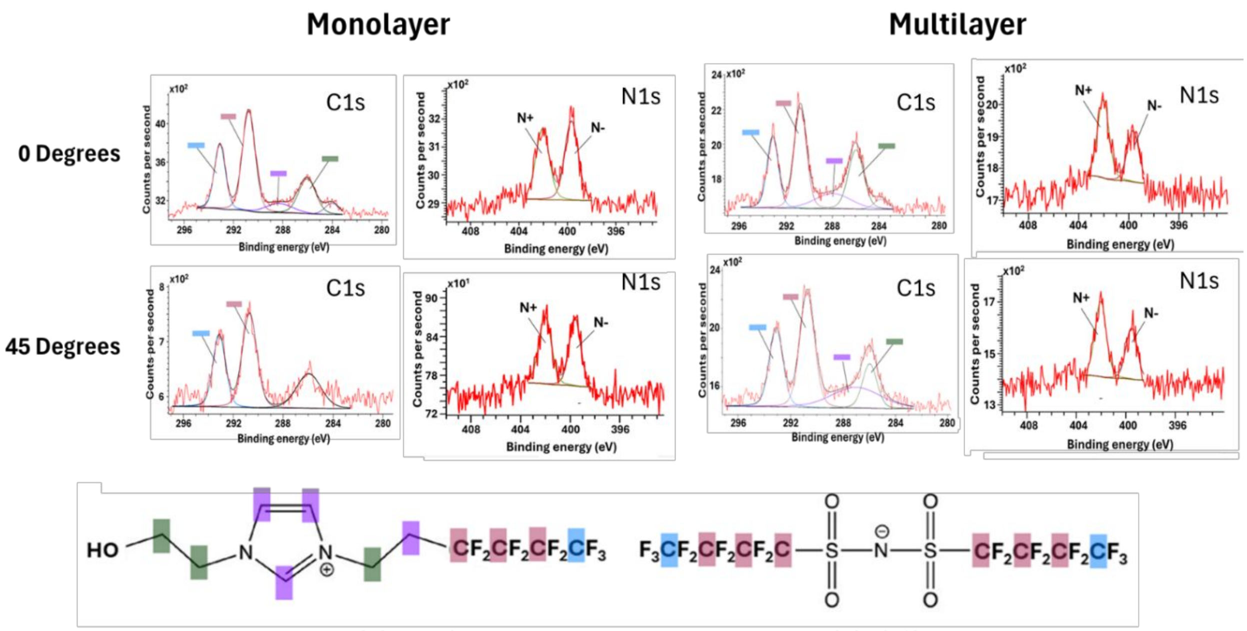

Figure S3: C1s and N1s ARXPS spectra for HFILOH on Si wafer at monolayer and multilayer thicknesses. C1s peaks are color-coded according to the molecular structure shown.

Table S1: Binding energies and peak areas for all assigned peaks in C1s and N1s XPS spectra shown in Figure 5

	Monolayer				Multilayer			
	0 Degrees		45 Degrees		0 Degrees		45 Degrees	
	Binding Energy (eV)	Peak Area	Binding Energy (eV)	Peak Area	Binding Energy (eV)	Peak Area	Binding Energy (eV)	Peak Area
N+	402.05	376.32	402.02	141.16	402.03	307.87	402.03	359.71
N-	399.67	421.65	399.62	132.40	399.66	199.87	399.53	243.82
CF_2	290.76	1444.85	290.80	768.61	290.74	839.87	290.73	1191.61
CF_3	293.10	795.37	293.14	477.37	293.11	537.15	293.11	751.35
C-OH, C-C	286.03	672.65	286.17	254.85	285.96	608.15	285.96	454.34
NCCN, NCN, CH_2-CF_2	288.32	280.74	288.33	238.86	287.96	371.14	287.02	738.60

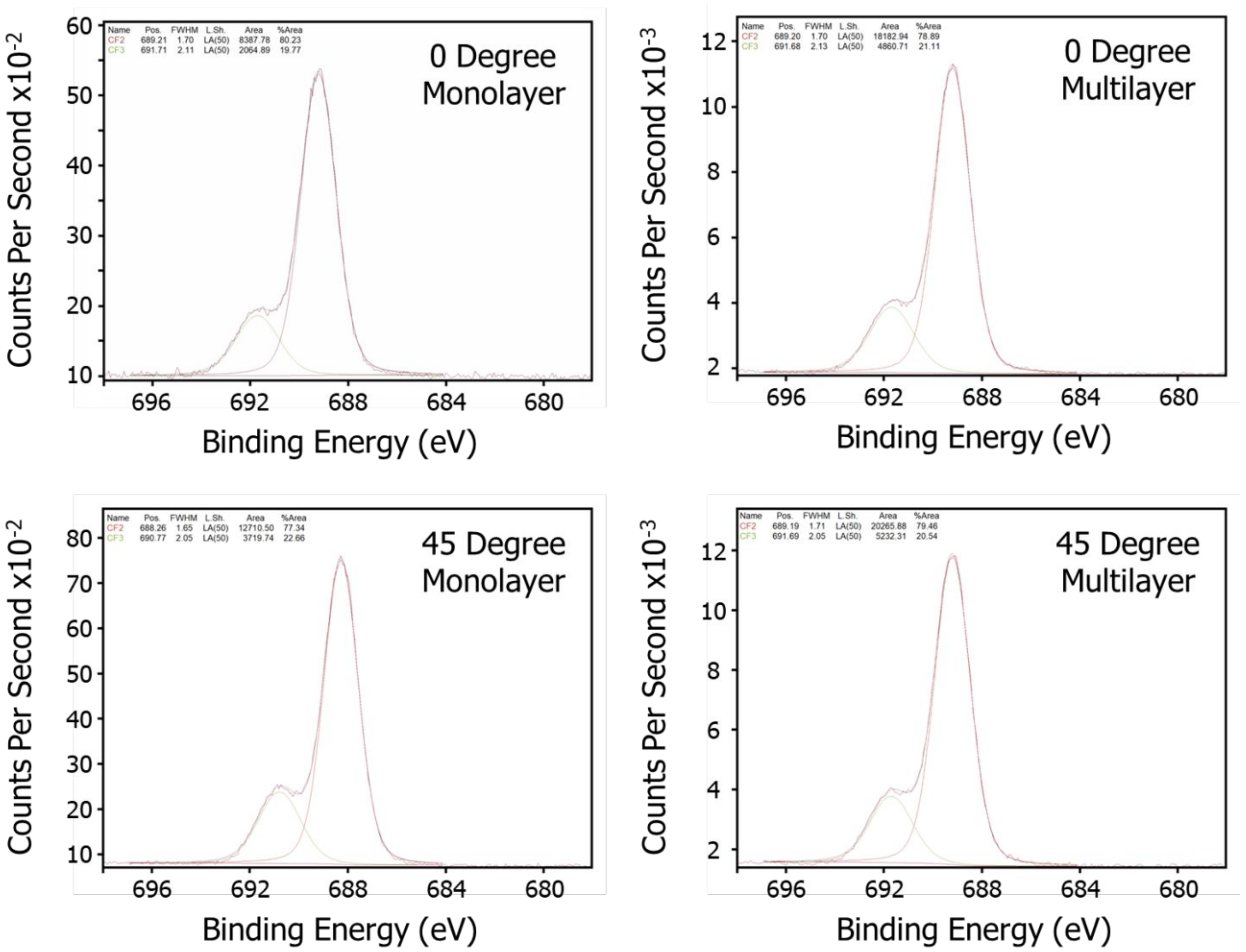

Figure S4: F1s XPS spectra of HFILOH coatings on silicon wafers at normal (0°) and angled (45°). Spectra are shown for both monolayer and multilayer thicknesses

Table S2: Binding energies and peak areas for assigned peaks in F1s XPS spectra

	Monolayer				Multilayer			
	0 Degrees		45 Degrees		0 Degrees		45 Degrees	
	Binding Energy (eV)	Peak Area	Binding Energy (eV)	Peak Area	Binding Energy (eV)	Peak Area	Binding Energy (eV)	Peak Area
CF_2	689.21	8387.78	688.26	12710.50	689.20	18182.94	689.19	20265.88
CF_3	691.71	2064.89	690.77	3719.74	691.68	4860.71	691.69	5232.31

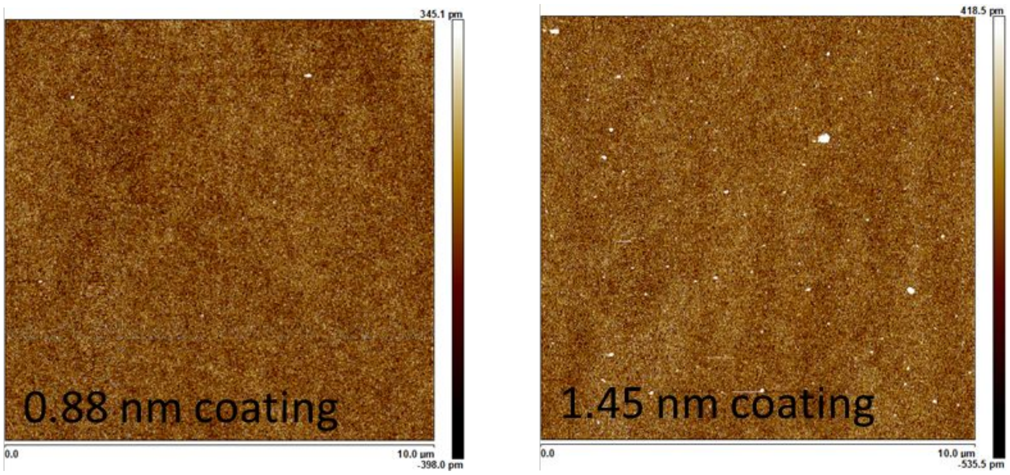

Figure S5: AFM for monolayer (left) and multilayer (right) HFILOH on Si wafer. Rq for monolayer is 0.146nm, and is 0.582nm for multilayer

Atomic Force Microscopy: The surface topography of the nanometer-thick ionic coating on Si wafer is evaluated by AFM using a Bruker Dimension Icon Atomic Force Microscope. The AFM probe is MikroMasch NSC14/AL BS with a resonance frequency of 160 kHz, a force constant of 5.0 N/m, and an 8 nm aluminum tip. The scans are conducted in tapping mode with an image acquisition of 512 × 512 pixels. The lateral resolution of the scans is 20 nm as the scan area is 10 × 10 μm^2, and the vertical resolution is ~0.1 nm
